# Expanded Indications for Neoadjuvant Endocrine Therapy in Early-Stage Breast Cancer During the COVID-19 Pandemic

**DOI:** 10.1245/s10434-024-15787-8

**Published:** 2024-08-12

**Authors:** Rhami Khorfan, Halley P. Vora, Jukes P. Namm, Naveenraj L. Solomon, Sharon S. Lum

**Affiliations:** Surgical Oncology Division, Department of Surgery, Loma Linda University Health, Loma Linda, CA USA

**Keywords:** Breast cancer, Neoadjuvant, Pandemic, Chemotherapy, Endocrine therapy, Surgery delay

## Abstract

**Background:**

In response to the COVID-19 pandemic, the Pandemic Breast Cancer Consortium (PBCC) published recommendations for triage of breast cancer patients. The recommendations included neoadjuvant treatment of early-stage breast cancer patients experiencing delays in surgery. This study evaluated national patterns of neoadjuvant treatment according to triage guidelines.

**Methods:**

Patients treated with surgery (upfront or post-neoadjuvant) in 2018–2020 were collected from the National Cancer Database. The proportions of patients treated according to the PBCC triage guidelines were calculated in 2020 and compared with similar cohorts in 2018–2019. Patient and hospital factors were evaluated for association with treatment.

**Results:**

Among cT1N0 ER+/PR+/HER2– patients, those treated in 2020 were more likely to receive neoadjuvant endocrine therapy (NET) compared with those before that time (odds ratio [OR], 3.08; range, 2.93–3.24). Among the patients with cT2N0 or cT1N1 disease, NET was more common in 2020 (OR, 1.76; range, 1.65–1.88). Academic facility, black or Asian race, more comorbidities, and the New England/Middle Atlantic region were associated with NET use.

**Conclusions:**

During the COVID-19 pandemic, expanded utilization of neoadjuvant therapy for surgical breast cancer patients was observed. Health care system limitations during the pandemic contributed to expanded adoption of neoadjuvant therapy for early breast cancer, contrary to usual practice. Long-term outcomes for patients treated according to PBCC recommendations should be closely monitored.

The COVID-19 pandemic posed enormous challenges to health care capacity and resources in 2020. The consequences of these challenges for cancer patients have been well documented, including decreases in screening and diagnosis, disruption of treatment, and projected negative impacts on patient outcomes.^1–4^ Because breast cancer is one of the most commonly diagnosed cancers in the United States,^5^ and because breast cancer surgery is among the most common cancer operations performed, the diagnosis and treatment of breast cancer may have been proportionally affected by the pandemic. Surgical capacity was significantly impacted in the pandemic, with hospitals in many areas forced to limit or delay elective operations during pandemic surges.^6–8^

To address these challenges, a group of surgical and cancer societies formed the Pandemic Breast Cancer Consortium (PBCC) to provide guidance to providers caring for breast cancer patients during the pandemic.^9^ This group published recommendations on triage of breast cancer patients based on different phases of the pandemic and hospital capacity. Recognizing that breast cancer surgery may be deemed “elective” and the subsequent delay in surgical treatment to which these patients would be subjected, the PBCC recommended consideration of neoadjuvant therapy for certain patient cohorts who would typically be treated with upfront surgery based on current guidelines.

Neoadjuvant endocrine therapy (NET), an important tool in the treatment of breast cancer, has accepted indications for patients with advanced disease and those who are not candidates for breast conservation.^10,11^ These patients typically have a higher T stage or node-positive disease. However, in the context of the pandemic, the indications for these treatments were expanded, and they were offered to patients with lower-stage early breast cancer.

Given the variation from accepted treatment paradigms imposed by the pandemic, our objectives were to evaluate national trends in the treatment of early breast cancer with neoadjuvant therapy in accordance to PBCC recommendations and to determine factors associated with use of neoadjuvant therapy for these patients.

## Methods

### Data Source and Patient Cohort

We performed a retrospective cohort study of the National Cancer Database (NCDB) from 2018 to 2020. The NCDB, a clinical oncology database jointly sponsored by the American College of Surgeons and the American Cancer Society, contains hospital registry data from more than 1500 facilities accredited by the Commission on Cancer (CoC). It represents more than 70 % of all cancers diagnosed in the United States, facilitating evaluation of treatment trends and associations on a national level.

Patients with a diagnosis of breast cancer from 2018 to 2020 were identified in the NCDB. The study excluded patients with clinical stage 0 or 4 disease and patients with unknown time from diagnosis to treatment because their treatment sequencing could not be determined. Only patients treated with surgical resection (whether upfront or after neoadjuvant therapy) were included in the analysis.

Using the PBCC triage guidelines, we identified two specific cohorts of patients defined by the PBCC as candidates for potential NET. These two cohorts included patients with clinical stage T1N0, ER+/PR+/HER2– and those with clinical T2 or N1, ER+/PR+/HER2–.^9^ We restricted our analysis only to these cohorts defined by the PBCC.

### Covariates

The analysis included and adjusted for several patient and hospital factors. The patient factors included age, categorized into decades (<30, 30–39, 40–49, 50–59, 60–69, 70–79, 80–89, and >90 years), and race/ethnicity, used evaluate possible differential impacts of the pandemic on minority populations and categorized as non-Hispanic white, non-Hispanic black, Asian, Hispanic, and other/unknown. Other socioeconomic factors included in the study were insurance status, quartile of median income by zip code, and urban/rural location. The Charlson-Deyo co-morbidity index was used to control for patient comorbidities.

The hospital factors included in the analysis were academic status and hospital geographic region, categorized by the NCDB into the following nine categories: New England, Middle Atlantic, South Atlantic, East North Central, East South Central, West North Central, West South Central, Mountain, and Pacific (see Appendix A for a full list of the states in each region).

### Statistical Analysis

The proportions of patients treated with neoadjuvant therapy according to the PBCC guidelines were calculated in 2020 and compared with similar cohorts from 2018 and 2019. Two pre-pandemic years were used to evaluate for secular trends in the use of neoadjuvant therapy during the time before the pandemic. Rates of neoadjuvant therapy were compared across years using the Cochran-Armitage test for trend. Logistic regression models were constructed to evaluate patient and hospital factors associated with reception of neoadjuvant treatment. This study was approved by the NCDB and found to be exempt from review by our institutional review board (IRB no. 5220326).

## Results

**Fig. 1:**
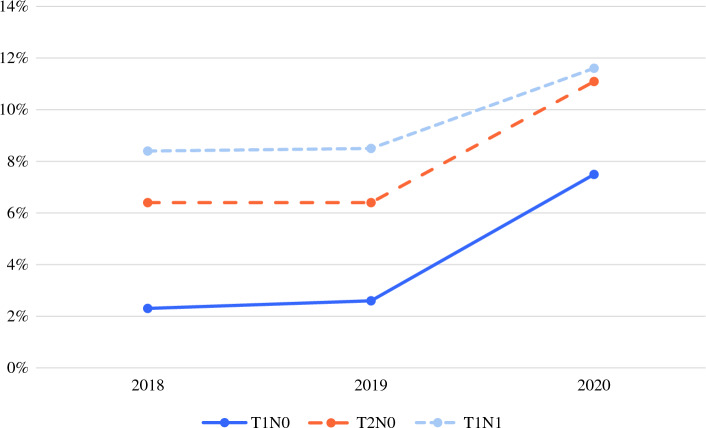
Trends in use of neoadjuvant endocrine therapy for ER+/PR+/HER2– breast cancer patients by T and N stage from 2018 to 2020. ER, estrogen receptor; PR, progesterone receptor; HER2, human epidermal growth factor receptor 2

The PBCC criteria for consideration of NET in 2020 were met by 87,857, including 68,435 T1N0, 16,845 T2N0, and 2577 T1N1 patients. Overall use of NET for eligible patients was 8.8 %, with higher use for patients with T2 or N1 disease (Table 1). The rates of NET utilization in the two pre-pandemic years were stable before increasing significantly in 2020 (from 2.6 % to 7.5 % for T1N0, and from 6.7 % to 11.2 % for T2 or N1; *p* < 0.01, trend test; Fig. 1).

**Table 1:** Neoadjuvant endocrine therapy by stage, patient, and hospital characteristics in 2020

	No neoadjuvant endocrine therapy		Neoadjuvant endocrine therapy		
	*n*	%	*n*	%	*p* value
*TNM stage cohort*					< 0.01
T1N0	63,083	92.2	5352	7.8	
T2N0	14,836	88.1	2009	11.9	
T1N1	2242	87.0	335	13.0	
*Age (years)*					< 0.01
18–29	179	85.2	31	14.8	
30–39	1966	90.4	209	9.6	
40–49	10,297	92.2	870	7.8	
50–59	16,526	91.6	1526	8.5	
60–69	24,450	91.2	2355	8.8	
70–79	20,315	91.3	1935	8.7	
80–89	5942	89.4	708	10.7	
>90	486	88.7	62	11.3	
*Race/ethnicity*					< 0.01
Non-Hispanic white	64,425	91.6	5883	8.4	
Non-Hispanic black	6,568	89.4	779	10.6	
Asian	3516	90.0	389	10.0	
Hispanic	3940	89.2	475	10.8	
Other/unknown	1712	91.0	170	9.0	
*Insurance status*					< 0.01
Not insured	774	88.9	97	11.1	
Private	37,205	91.8	3326	8.2	
Medicaid	4008	89.3	481	10.7	
Medicare	36,674	91.0	3647	9.0	
Other government	900	91.0	89	9.0	
Unknown	600	91.5	56	8.5	
*Education quartile*					0.01
Q1	10,528	90.6	1099	9.5	
Q2	17,213	91.4	1626	8.6	
Q3	20,761	91.5	1926	8.5	
Q4	18,882	91.6	1735	8.4	
*Median income quartile*					0.01
Q1	8271	91.2	802	8.8	
Q2	13,329	92.0	1160	8.0	
Q3	16,144	91.5	1506	8.5	
Q4	29,513	91.0	2906	9.0	
*Urban/rural residence*					< 0.01
Metropolitan area	67,932	91.0	6759	9.1	
Non-metro urban	9527	93.1	708	6.9	
Rural	1145	93.2	83	6.8	
*Charlson comorbidity score*					< 0.01
0	65,052	91.5	6072	8.5	
1	10,235	90.7	1052	9.3	
2	2884	90.0	320	10.0	
3	1990	88.8	252	11.2	
*Academic hospital*					< 0.01
No	55,679	92.3	4616	7.7	
Yes	22,337	88.7	2840	11.3	
*Hospital location*					< 0.01
New England	4960	89.9	558	10.1	
Middle Atlantic	10,932	89.6	1263	10.4	
South Atlantic	18,089	92.7	1426	7.3	
East North Central	13,501	90.9	1347	9.1	
East South Central	4933	90.9	492	9.1	
West North Central	6251	94.1	393	5.9	
West South Central	5780	89.5	680	10.5	
Mountain	3831	92.8	298	7.2	
Pacific	9739	90.7	999	9.3	

TNM, tumor-node-metastasis

### Patient and Hospital Factors Associated with Neoadjuvant Therapy

**Fig. 2:**
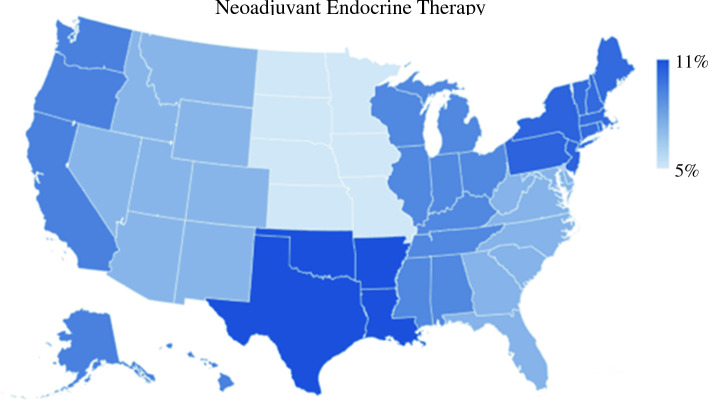
Regional variation in use of neoadjuvant endocrine therapy according to Pandemic Breast Cancer Consortium (PBCC) triage guidelines in 2020

Several patient and hospital factors were associated with neoadjuvant treatment in the unadjusted analysis (Table 1). The patients at the extremes of age (age 18–29 years or >80 years) and those with more co-morbidities were more likely to be treated with NET. There were socioeconomic differences in receipt of neoadjuvant therapy, including higher NET use for black (10.6 %) and Hispanic (10.8 %) patients than for white patients (8.4 %), and also higher NET use for uninsured patients (11.1 %) and Medicaid (10.7 %) patients than for those with private insurance (8.2 %). Academic hospitals were more likely to provide neoadjuvant treatment than non-academic facilities. Hospital location also was associated with neoadjuvant therapy use, with significant variation by geographic region (Fig. 2).

In the multivariable logistic regression analysis, the factors that remained significantly associated with NET use were age older than 80 years (odds ratio [OR], 1.27; range, 1.12–1.43; *p* < 0.01), black race (OR, 1.26; range, 1.15–1.39; *p* < 0.01), high Charlson comorbidity score (OR, 1.29; range, 1.11–1.51; *p* = 0.02), academic facility (OR, 1.49; range, 1.41–1.58; *p* < 0.01), and geographic regions including the Middle Atlantic (OR, 1.35; range, 1.23–1.47; *p* < 0.01), New England (OR, 1.39; range, 1.23–1.56; *p* = 0.01), Pacific (OR, 1.39; range, 1.26–1.53; *p* < 0.01), West South Central (OR, 1.49; range, 1.33–1.65; *p* < 0.01) regions (Table 2).

**Table 2:** Multivariable logistic regression of neoadjuvant endocrine therapy use for patients meeting the pandemic breast cancer consortium criteria

	OR	95 % CI		*p* value
*Age (years) (reference = 50–59)*				
40–49	0.91	0.83	1.00	**< 0.01**
60–69	1.06	0.97	1.15	0.51
70–79	1.03	0.93	1.14	0.19
80–89	1.27	1.12	1.43	**< 0.01**
>90	1.27	0.92	1.75	0.22
*Charlson (reference = 0)*				
1	1.06	0.98	1.14	0.10
2	1.16	1.02	1.33	0.51
3	1.29	1.11	1.51	**0.02**
*Race/ethnicity (reference = non-Hispanic white)*				
Non-Hispanic black	1.26	1.15	1.39	**< 0.01**
Asian	1.09	0.96	1.23	0.75
Hispanic	1.20	1.07	1.35	0.10
Other/unknown	1.00	0.83	1.19	0.17
*Insurance status (reference = private)*				
Not insured	1.22	0.95	1.57	0.43
Medicaid	1.15	1.02	1.30	0.65
Medicare	1.06	0.99	1.15	0.34
Other government	1.16	0.90	1.49	0.74
Unknown	1.12	0.82	1.52	0.99
*Income quartile (reference = Q4)*				
Q1	0.98	0.89	1.08	0.97
Q2	0.95	0.88	1.03	0.23
Q3	0.99	0.93	1.06	0.64
*Urban/rural residence (reference = metropolitan area)*				
Urban, non-metropolitan	0.84	0.76	0.92	0.49
Rural	0.78	0.60	1.03	0.26
Academic (reference = no)	1.49	1.41	1.58	**< 0.01**
*Hospital location (reference = South Atlantic)*				
East North Central	1.25	1.15	1.37	0.32
East South Central	1.34	1.19	1.52	**0.05**
Middle Atlantic	1.35	1.23	1.47	**< 0.01**
Mountain	1.07	0.93	1.24	**0.05**
New England	1.39	1.23	1.56	**0.01**
Pacific	1.39	1.26	1.53	**< 0.01**
West North Central	0.82	0.72	0.93	**< 0.01**
West South Central	1.49	1.33	1.65	**< 0.01**

OR, odds ratio; CI, confidence interval

## Discussion

In this retrospective observational study of a national database, the use of neoadjuvant therapy for early breast cancer increased during the pandemic. This increase was in concordance with the cohorts defined by breast cancer societies and outlined in the PBCC recommendations for cancer triage in response to resource limitations imposed by the pandemic in 2020. The observed increase was particularly pronounced among patients with the lowest disease burden, with a threefold increase in NET for T1N0 patients. This finding is notable because the standard management for these patients before the pandemic and according to current guidelines was upfront surgical treatment.

The role of NET for hormone receptor-positive cancer has been the subject of numerous prospective studies, with varying rates of clinical and pathologic response.^12–15^ The Z1031 trial was a phase 2 study of neoadjuvant endocrine therapy for postmenopausal stages II and III estrogen receptor-positive (ER+) patients who demonstrated a clinical response rate of 63 % to 75 % for NET, a breast-conserving surgery (BCS) rate of 68 %, and significant Ki-67 suppression of 78–87 %.^14^

In a phase 3 study comparing NET and neoadjuvant chemotherapy (NACT) for pre-menopausal patients with estrogen receptor-positive (ER+) N+ breast cancer, NET demonstrated a lower clinical response (53 % vs 84 %), as well as a lower nodal pathologic complete response (pCR) (4.9 % vs 13.8 %) than NACT.^15^ However, BCS conversion rates and change in Ki-67 expression did not differ significantly between the two groups. The authors also noted that the adverse event rate was significantly lower in the NET group.

A retrospective review of NCDB data similarly demonstrated lower rates of pCR for NET than for NACT, although the data highlighted that some degree of T (40.5 %) and N (18.3 %) downstaging still was attainable using NET.^13^ Although NET demonstrated significant clinical response, conversion of BCS-ineligible or nonoperative patients to BCS, and Ki-67 suppression, the rates for pathologic complete response (pCR) were consistently low, particularly compared with those for NACT. Despite this difference, the clinical benefits of NET make it a valuable tool in the treatment of hormone-positive breast cancer.

An added benefit of NET may be its role in prognosis and prediction of response to chemotherapy, which can help guide adjuvant treatment. In an analysis of the P024 neoadjuvant endocrine trial, tumors from 228 postmenopausal ER+ stages 2 and 3 patients were analyzed for posttreatment ER status, Ki-67, grade, tumor size, nodes, and treatment response. The findings showed a highly significant association between post-treatment Ki-67 and recurrence-free survival. The results suggest that for patients with a low pathologic stage and a favorable biomarker profile, recurrence rates were so low that adjuvant systemic therapy may be unnecessary.^16^

A recent phase 3 study aimed to directly address this question of whether patients responding to NET should receive endocrine therapy alone or endocrine and chemotherapy as adjuvant therapy. The results suggested that adjuvant chemotherapy may not be needed for patients with clinical T1c or a low proliferative index who demonstrate response to NET.^17^ Although not the sole determining factor, response to NET may help inform the decision on adjuvant chemotherapy, but further study is needed to clarify its role.

It is important to note that the majority of the existing evidence stems from studies investigating the clinical applications of NET for patients with T2 or node-positive disease, with exclusion of patients who had T1N0 disease. However, the largest increase in NET use found in our study was among the T1N0 patients, suggesting that this increase was not necessarily in response to emerging evidence and more likely was due to logistical and resource-related factors associated with the pandemic. Our findings are corroborated by a recent survey study of 114 surgical, medical, and radiation oncologists who found that 91 % of providers reported changing their practice due to the pandemic, including the use of NET as initial treatment.^18^

A prior NCDB analysis found that NET was used for 2.7 % of Z1031-eligible patients during the pre-Z1031 era compared with 3.2 % of these patients after Z1031.^19^ This report is consistent with our findings and highlights the large increase in NET use nationally in 2020, up to 7.5 % for T1N0 and 11.2 % for T2/N1. A single-institution study of 573 patients found even higher adoption of NET during the pandemic, from 7 % before the pandemic to 16 % for all patients, and from 7 % to 22 % specifically for clinical stage I HR+/HER2– patients.^20^

The observed increase in NET during the pandemic can serve as an important natural experiment on the role of NET in this cohort of patients, and outcomes for these patients should be closely monitored during subsequent years to potentially guide future prospective studies.

Racial and ethnic disparities in the use of neoadjuvant therapy were seen during the pandemic. The underlying reason why black and Asian patients were more likely to receive NET is unclear and cannot be further explored given the limitations of the NCDB. Possible explanations include differential impacts of the pandemic on minority populations, contributing to less access to surgical care, but the role of patient or provider decision-making, implicit bias, or structural racism cannot be examined. Differences in the individual-level impact of the pandemic on minorities as well as the hospital-level impact on minority-serving institutions likely contributed to the disparities.^21,22^

Regional differences in the use of NET reflect interesting parallels to the early waves of the pandemic. Although not an exact match, the higher use of NET in the Middle Atlantic region corresponds to the peak in the first wave of the pandemic, whereas the South Atlantic and Southern regions bore the brunt of the second wave.^23^ These findings support the notion that the increases in NET use likely were linked to impacts of the pandemic.

Our study should be interpreted in light of certain limitations, most notably that this study was retrospective and subject to inherent selection bias. The underlying reason or intent in choosing neoadjuvant therapy cannot be determined, and attributing its increased use to the pandemic is an unverifiable assumption. In addition, the NCDB provides data only from CoC-accredited institutions, meaning that that it does not account for patients treated at other, likely smaller, hospitals. Furthermore, when the impact of the pandemic on patients is discussed, it is important to note that those who experienced the most negative impact of the pandemic likely never presented to a hospital, and therefore would not be included in any database. Despite these limitations, our results demonstrate a clear trend of neoadjuvant treatment expansion during the first year of the pandemic.

National data suggest that providers responded to the challenges of the pandemic by implementing society recommendations for neoadjuvant treatment. These findings are encouraging in that potential negative patient outcomes resulting from the pandemic may have been mitigated to some extent. Unfortunately, the sobering negative impact of the pandemic on cancer survival has been projected in multiple studies.^1,24^ Therefore, the outcomes for these patients should be closely monitored to ensure appropriate care and follow-up assessment, as well as to inform not only responses to future challenges, but also prevailing treatment paradigms, with an eye toward further refinement and improvement.

## Appendix A

List of states included in each geographic region

**Table:**

Region	States
New England	CT, MA, ME, NH, RI, VT
Middle Atlantic	NJ, NY, PA
South Atlantic	DC, DE, FL, GA, MD, NC, SC, VA, WV
East North Central	IL, IN, MI, OH, WI
East South Central	AL, KY, MS, TN
West North Central	IA, KS, MN, MO, ND, NE, SD
West South Central	AR, LA, OK, TX
Mountain	AZ, CO, ID, MT, NM, NV, UT, WY
Pacific	AK, CA, HI, OR, WA
